# Nanobiomaterial-enabled boron delivery systems and innovative strategies for revolutionizing BNCT

**DOI:** 10.7150/thno.126209

**Published:** 2026-02-11

**Authors:** Qiyao Yang, Ruxuan Wang, Huang Lingling, Qiong Bian, Qichun Wei

**Affiliations:** 1 Department of Radiation Oncology, Key Laboratory of Cancer Prevention and Intervention, The Second Affiliated Hospital, College of Medicine, Zhejiang University, Hangzhou 310058, Zhejiang, China.; 2 Department of Pharmacy, Sir Run Run Shaw Hospital, Zhejiang University School of Medicine, Hangzhou 310016, Zhejiang, China.; 3 Department of Pharmacy, The Second Affiliated Hospital, Zhejiang University School of Medicine, Hangzhou 310009, China.; 4 Center for Plastic & Reconstructive Surgery, Department of Dermatology, Zhejiang Provincial People's Hospital, Affiliated People's Hospital, Hangzhou Medical College, Hangzhou 310014, Zhejiang, China.

**Keywords:** boron neutron capture therapy (BNCT), drug delivery, nanotechnology, multifunctional nanostructures, tumor targeting, biological mechanism

## Abstract

Boron neutron capture therapy (BNCT) is a novel and emerging form of radiotherapy that combines the advantages of “heavy ion radiotherapy” and “biological targeting”. The therapeutic potential of BNCT is ultimately constrained by precise and efficient delivery of boron drugs. In this review, we systematically trace from conventional boron drugs to sophisticated nano-delivery platforms, emphasizing breakthroughs in carrier engineering, targeted delivery strategies, and multifunctional synergistic systems. A dedicated analysis of the biological foundations, such as cell cycle dynamics, tumor microenvironmental interactions, and immunostimulatory effects, provides a crucial framework for understanding mechanism-informed biomaterial design. By synthesizing current advances with an outlook on future challenges, this work aims to chart a course for translating innovative boron-loaded biomaterials from the laboratory into clinical reality, thereby unlocking the full promise of BNCT.

## 1. Introduction

Cancer is one of the leading causes of death, with an estimated 20 million new cases and 9.7 million deaths worldwide in 2022, which will become a major stumbling block on the road to health for humans^1^. Radiotherapy is a kind of local and noninvasive treatment that kills tumors by radiation, which is relatively safer than chemotherapy and more applicable than surgery. However, its background radiation can inflict damage upon adjacent normal tissues. And the therapeutic effect is limited by the tumor's hypoxic environment.

In recent years, radiotherapy technology has undergone a progressive transition from traditional, single-modality approaches to more precise and comprehensive strategies^2^. Stereotactic body radiotherapy (SBRT) and particle radiotherapy have significantly enhanced treatment efficacy and safety^3^. Particles with higher linear energy transfer (LET) radiation cause DNA damage to cells that is harder to repair, yielding a higher relative biological effectiveness (RBE). Moreover, particle radiotherapy can partially circumvent the limitations imposed by tumor hypoxia, which makes it have a low oxygen enhancement ratio (OER)^4^-^8^. Consequently, particle radiation can efficiently destroy hypoxic tumors, which are resistant to conventional radiotherapy^9^, and it has already been implemented in clinical practice^10^-^12^.

Boron neutron capture therapy (BNCT) is emerging as a rising star among particle radiotherapy. Following the discovery of neutrons by the British physicist James Chadwick in 1932, the famous theory of the boron neutron capture reaction was proposed by Tylor and Goldhaber in 1935^13^, which laid the groundwork for BNCT. In 1936, Gordon Locher first proposed the application of the boron neutron capture reaction to tumor therapy^14^.

^10^B atoms possess a large thermal neutron capture cross section (3850 barns, 1 barn = 10^-24^ cm^2^) and can undergo fission reactions to produce alpha rays (^4^He) and lithium particle rays (^7^Li) after capturing uncharged thermal neutrons emitted from the outside. They are all high LET particle beams with significantly higher RBE than X-rays and γ-rays used in conventional tumor radiotherapy^15^. Moreover, the range of them in the tissue is only 5-9 μm, which limits the high destructive energy to a single cell^16^.

The implementation of BNCT relies on the delivery of ^10^B-containing drugs to the tumor sites. The intervention of the boron drug offers the chance to connect radiotherapy with targeted therapy. Therefore, BNCT possesses the dual targeting advantages of the targeting contouring of radiotherapy and the potential targeting ability of drug delivery.

By combining "biological targeting" and "heavy ion radiotherapy", BNCT can reduce toxic effects and enhance the killing effect on tumor tissue, holding promise to revolutionize the paradigms of cancer treatment. Therefore, numerous clinical trials^17^,^18^ related to BNCT have been conducted, and it has been applied in the treatment of various malignancies, including head and neck tumors, melanomas, glioblastomas, oral cancer, and recurrent tumors^19^-^26^.

## 2. Boron Drugs

Apart from a stable, pure, and high-intensity thermal neutron source for BNCT development, a prerequisite for this dual-modality treatment to achieve maximum benefit is the precise delivery of the ^10^B into the tumor cells and maximizing the difference in boron content between normal and tumor cells.

**Figure 1:**
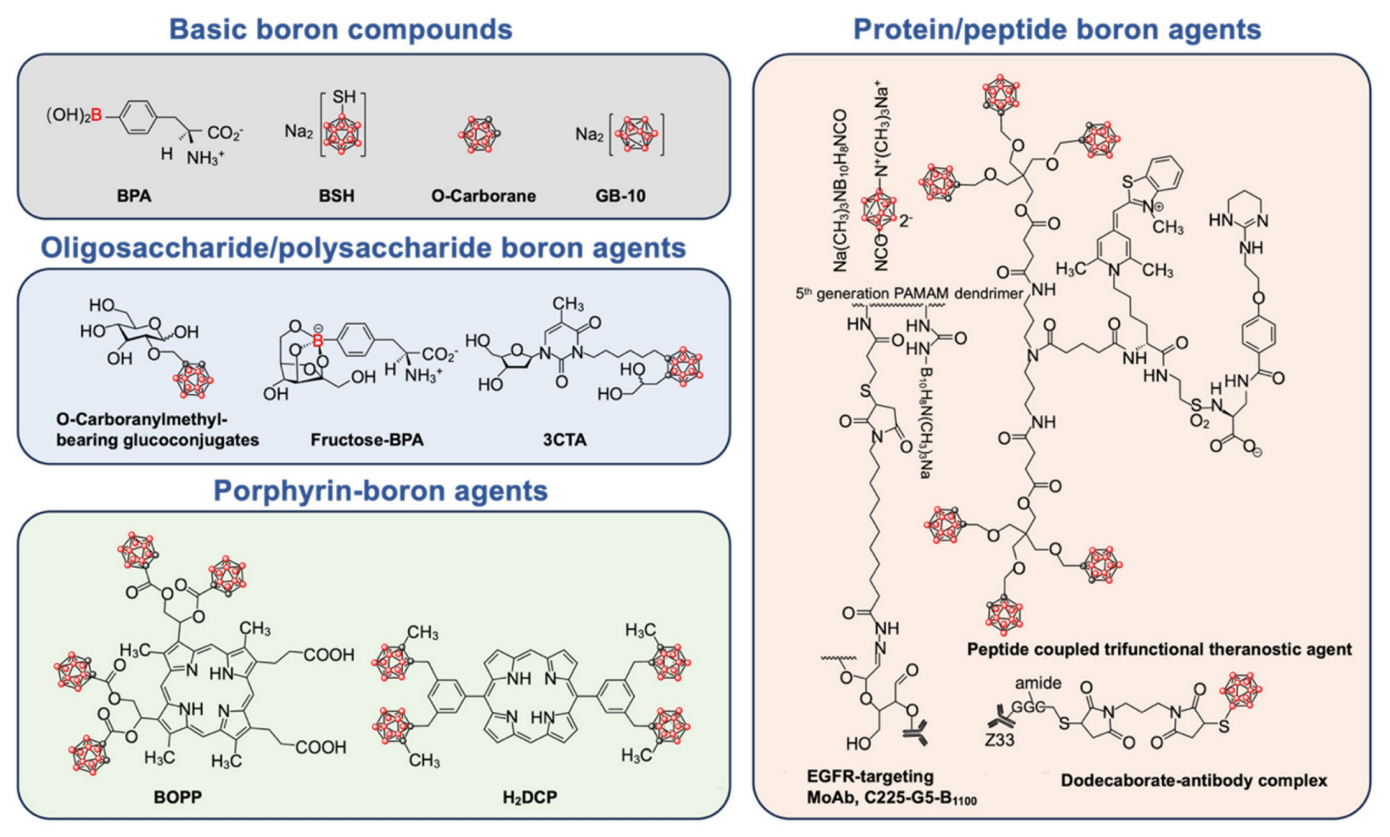
Schematic diagram of chemical structure of basic boron drugs and their derivatives. The boron element is represented by red spheres.

The ideal boron drug must fulfill the following criteria: 1) achieving a tumor boron-10 concentration within the range of approximately 20-50 μg/g; 2) exhibiting selective boron concentration ratios in tumor versus normal tissue (T/N) and tumor versus blood (T/B) that are greater than 1, with a preference for 3 or higher; and 3) possessing minimal systemic cytotoxicity, rapid clearance from the bloodstream and normal tissues, while maintaining retention within the tumor during neutron irradiation. Therefore, boron drugs have undergone several generations of transformation (Figure 1) and are gradually evolving towards nanotechnology.

### 2.1. Basic boron drugs

Several boron drugs have been developed for possible use in BNCT, but only two, boronophenylalanine (BPA) and sodium borocaptate (BSH), have been clinically tested. Another boron drug with great application potential in BNCT is boron cluster, which has a high boron content. Many derivatives of these boron drugs have been developed in order to overcome their limitations.

#### 2.1.1. Sodium borocaptate (BSH)

In 1967, Soloway and Hatanaka proposed a polyhedral borane anion named BSH with the chemical structural formula Na_2_B_12_H_11_SH^27^. Hatanaka used it for BNCT of glioblastoma multiforme (GBM) in 1968^28^. BSH is a hydrophilic small molecule with high boron content, capable of transporting 12 boron atoms per molecule. According to a European phase I clinical study (EPRTC11961), BSH had a brain tissue-to-blood boron ratio of 0.2 ± 0.02 and a tumor-to-blood boron ratio of 0.6 ± 0.2^29^. However, it lacks active targeting capability. Furthermore, due to its net charge, it is unable to cross the cell membrane, limiting its practical application.

#### 2.1.2. Boronophenylalanine (BPA)

In 1968, Snyder *et al.* synthesized a neutral amino acid phenylalanine derivative called BPA^30^. A clinical trial of BPA in malignant melanoma was conducted in 1987 by Mishima *et al.*^31^. In patients with malignant melanoma, Fukuda *et al.* reported a mean skin-to-blood boron ratio of 1.31 ± 0.22 and a tumor-to-blood boron ratio of 3.40 ± 0.83 for BPA^32^.

Research has found that BPA can be actively transported into cells *via* the L-type amino acid transporter system (LAT-1), which is highly expressed in tumor cells^33^. Therefore, BPA is generally preferred over BSH in clinical applications. However, BPA's water solubility is relatively low, at around 1.6 g/L, necessitating its formulation as a fructose complex (BPA-f) to improve its hydrophilicity. Furthermore, because BPA contains only one boron atom per molecule, it takes extremely high doses (500 mg/kg) to achieve the desired boron concentration in tumors. The hydrophobicity and low boron content restrict its tumor specificity^34^.

#### 2.1.3. Carboborane

Boron exhibits similarities to carbon in its ability to form covalent boron-hydrogen bonds and compounds with boron-boron interactions. To compensate for electron deficiencies, boranes tend to form polyhedral clusters with spherical structures. However, most boranes are unstable in aqueous environments. Carborane, the new compound formed by replacing two BH units with CH vertices, has exceptional biostability. Moreover, the two carbon atoms can serve as a foundation for organic modifications^35^.

Carborane is structurally similar to the benzene ring in three dimensions, making it a unique pharmacophore. It has a high boron atomic content and a high neutron capture cross-section, making it an excellent candidate for BNCT^36^. As a result, carboranes have been extensively studied for modification with nanoparticles, antibodies, peptides, enzyme/receptor inhibitors, glycans, and other substances. Many chemists are still investigating various chemical modification processes for carboranes^37^,^38^. As a result, carborane will be an advantageous boron drug in BNCT's upcoming multifunctionalization process.

### 2.2. Derivatives of boron drugs

Existing boron drugs are limited by poor solubility, weak biocompatibility, and inadequate targeting capabilities. To address these limitations and expand their applications, researchers have modified boron drugs with various bioactive organic compounds, leading to the development of boron drug derivatives, including nucleoside/nucleotide boron drugs, protein/peptide boron drugs, oligosaccharide/polysaccharide boron drugs, and porphyrin-boron drugs.

These boron derivatives offer distinct advantages due to their varied organic compounds. For instance, nucleoside/nucleotide boron drugs combined with siRNA or ASOs can integrate BNCT with gene therapy. Protein/peptide boron drugs utilize functional proteins or peptides to achieve targeted delivery to tumors and realize some biological effects. Oligosaccharide/polysaccharide boron drugs enhance water solubility and biocompatibility, enabling tumor targeting through sugar transporters. Porphyrin-boron drugs exploit the photosensitizing properties of porphyrins to combine BNCT with photodynamic therapy (PDT). The specific functionalities of these boron drug derivatives will be discussed in detail in the following sections.

## 3. Boron-Containing Nano-System

Nanoparticles have gained significant attention in the field of drug delivery due to the enhanced permeability and retention (EPR) effect and their structural advantages. In BNCT, nanoparticles have been widely utilized to achieve passive-targeted delivery of boron drugs to tumors, enhance their water solubility and biocompatibility, increase boron loading, enable controlled release, and facilitate the functionalization of boron drugs. Therefore, in recent years, nano-delivery systems based on different materials have been extensively employed in BNCT^39^. In addition to a summary of boron delivery nano-systems, personalized delivery and treatment strategies based on the specialty of BNCT are presented in this review.

### 3.1. Increase tumor targeting delivery of boron (Initial development trend)

Many antineoplastic drugs cause significant physical and psychological harm to patients because they destroy both healthy and cancerous tissues non-selectively. So as to boron drugs, which can cause radiation damage to healthy tissues after neutron capture. From the perspectives of safety and efficacy, the initial development trend of the boron delivery system was to realize tumor-targeting delivery. Traditional mechanisms for tumor-targeted delivery primarily include the EPR effect of nano-systems, ligand-receptor interactions, and stimuli-responsive drug release mechanisms.

#### 3.1.1. The EPR effect of nano-systems

In 1986, Matsumura and Maeda proposed the EPR effect, which has become a guiding principle for the development of nanomedicine. During tumor angiogenesis, the interstitial spaces between vascular endothelial cells can exceed 200 nm, in contrast to the tightly arranged endothelial cells in normal tissues^40^,^41^. This structural difference allows nanometer-sized particles to penetrate the tumor microenvironment more effectively, while limiting their access to normal tissue microenvironments. And the size of these particles is typically large enough to avoid renal clearance. While at the tumor site, impaired lymphatic drainage further hinders the clearance of particles, contributing to their retention within the tumor^42^.

Nanotechnology has witnessed significant advancements in the field of drug delivery, which has also made substantial contributions to the delivery of boron drugs in BNCT. A variety of nanoscale structures have been successfully employed for this purpose, including nanoparticles^43^-^46^, micelles^47^-^51^, liposomes^52^,^53^, boron nitride nanotubes^54^, and dendritic polymers^55^. Nanoplatforms of different materials exhibit different advantages. Inorganic nanoparticles are readily accessible, amenable to large-scale preparation, and have better stability and higher boron-loading capacities. Their intrinsic superparamagnetism and fluorescence further enhance their theranostic capability. Conversely, organic nanoparticles display lower cytotoxicity and immunogenicity, and their surfaces can be conveniently functionalized with ligands to achieve enhanced targeting and membrane-penetrating efficiency. Consequently, both inorganic and organic materials possess unique advantages and have demonstrated promising performance in BNCT.

#### 3.1.2. Ligand-receptor interactions

Active targeting, which is mediated by ligand-receptor interactions, can be used in addition to passive targeting. As a result, many target ligand-modified boron drugs have been developed for BNCT, which are listed in Table 1.

**Figure 2:**
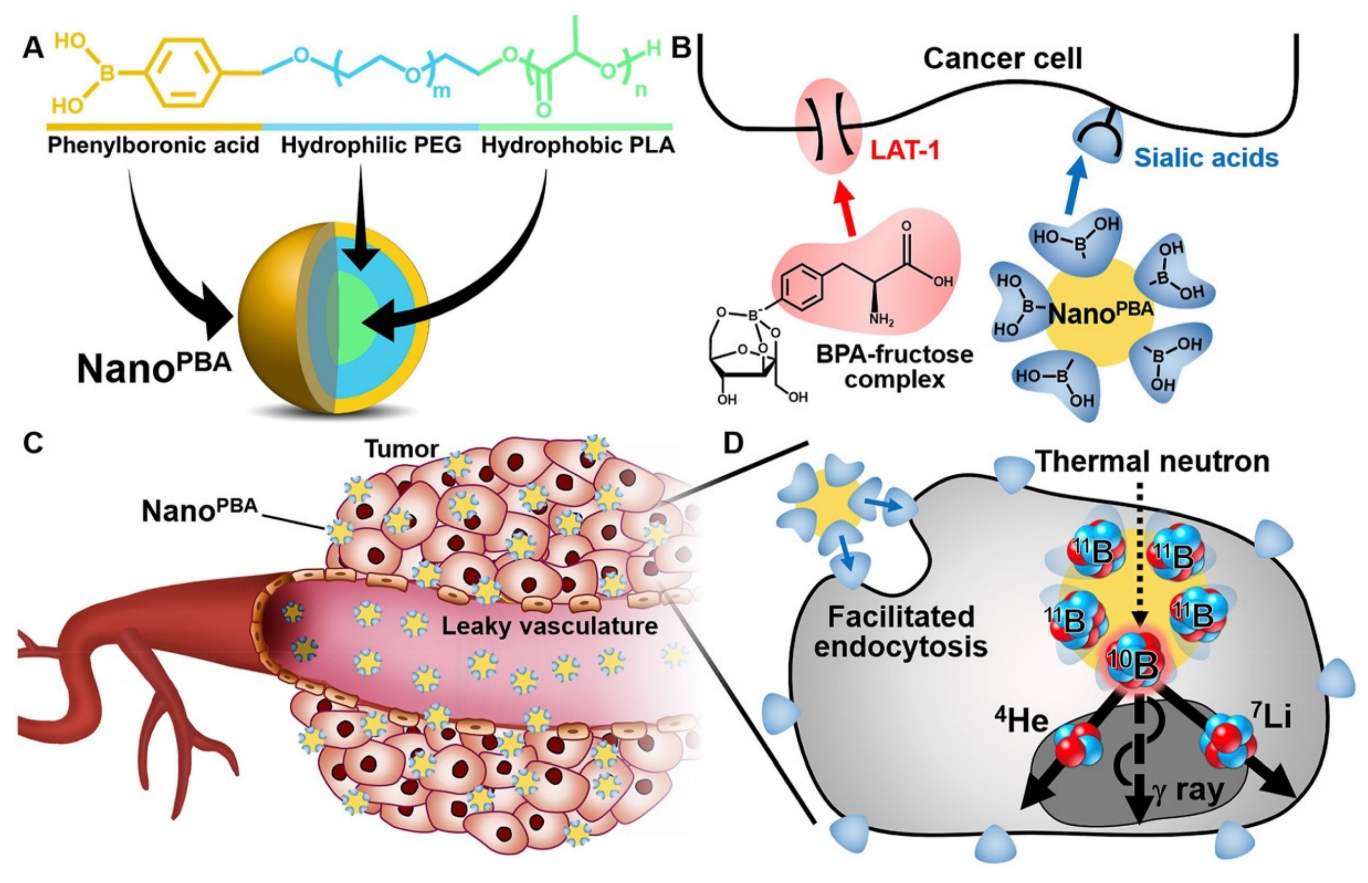
Schematic illustrations of PBA modified polymers^59^, copyright 2020 Elsevier. A. Chemical structure and molecular design of the Nano^PBA^. B. Different mechanisms of cellular uptake facilitation between BPA-fructose complex and Nano^PBA^. C. Passive targeting of the tumors via the EPR effect. D. Facilitated endocytosis of the Nano^PBA^ and subsequent nuclear fission in response to the thermal neutron irradiation.

BPA is a specialized ligand used in BNCT, serving as both a source of boron and a targeting moiety for tumor cells due to the overexpression of its receptor, LAT-1, on tumor cells^56^,^57^. BPA-modified mesoporous silica nanoparticles have been utilized to enhance the boron loading capacity and the accuracy of targeted delivery^58^. Besides, boric acid derivatives have been identified as another promising boron-containing ligand for targeted drug delivery due to their selective binding affinity for salivary acid (SA)^59^. Ahram Kim *et al.* modified phenylboronic acid (PBA) on PLA-PEG polymers, achieving a potent anti-tumor effect with a dosage 100 times lower than that of BPA-fructose (BPA-f) (Figure 2)^59^.

Boron-containing ligands represent a specialized subset of ligands used for boron delivery to tumors. Many classical ligands have also been employed for this purpose. For instance, the folate receptor (FR) is overexpressed in several types of human cancers, including ovarian, brain, renal, breast, myeloid, and lung malignant tumors^60^,^61^. Folate-modified nanostructures can be specifically internalized *via* endocytosis through FR^62^. Studies have reported folic acid modification of boron nitride nanoparticles^57^, boron carbide nanoparticles^63^, boron phosphate nanoparticles^62^, iron-containing nanoparticles^64^, liposomes^53^, and PLGA nanoparticles^65^.

Carbohydrates can also be used to increase the delivery accuracy of boron to tumor sites. Leveraging the Warburg effect, D-glucose derivatives containing o-carborane have been designed to target GLUT1 overexpressed in tumors^66^,^67^. Carbohydrates play a role in a variety of biological processes in addition to interacting with different cell-surface proteins to achieve targeted delivery. For instance, the interaction between galactose and asialoglycoprotein receptors (ASGP-Rs) on HepG_2_ cells can trigger the receptor-mediated endocytosis. Therefore, galactose-grafted carboranes can improve boron delivery to HepG_2_ cells and increase cell-killing efficacy by tenfold^68^. Besides, the high affinity of hyaluronic acid (HA) for CD44, which is overexpressed on tumor cells, was utilized to create a tumor-targeting hydrosoluble complex of HA and o-carborane for boron delivery. The complex's efficacy can surpass that of the BPA-fructose complex, which has widespread clinical use^69^.

The antibody-drug conjugate (ADC) has gained significant momentum due to its targeted delivery, achieved by interacting with specific receptors. These coupling tactics have also been employed in BNCT. One early study has combined boron with cetuximab, a chimeric monoclonal antibody that targets EGFR, achieving not only the specific delivery of boron to gliomas but also exerting immunotherapeutic effects by blocking downstream signaling pathways^70^-^72^. Another study combined an anti-HER_2_ antibody (61 IgG) with boron-containing polyethylene glycol-coated gold nanoparticles, resulting in a significant increase in the accumulation of boron-containing nanoparticles in HER_2_-overexpressing tumors through phagocytosis^73^.

However, the large molecular weight and immunogenicity of antibodies restrict their application. Therefore, a small molecule peptide-drug conjugate (PDC) has emerged as a promising alternative to mitigate the drawbacks. These peptides can be categorized into two main types: cell-penetrating peptides (CPPs) and cell-targeting peptides (CTPs). RGD, one of the CTPs, as a tumor-homing peptide can be utilized to modify boron drugs directly, as well as grafted onto a boron nano-delivery system. RGD peptides specifically recognize and bind to integrin receptors (such as αvβ3 and αvβ5) that are overexpressed on tumor cells (breast cancer, glioma, pancreatic tumors, etc.) and tumor-associated endothelial cells^74^. Various RGD-modified nano-systems have demonstrated enhanced tumor penetration and accumulation^73^-^75^. In our team's iRGD-modified polymer study, the nanostructures achieved a T/B ratio of 14.11 and a T/N ratio of 19.49 *in vivo*^75^.

Ligand modification represents the most direct approach for nano-systems to target tumors, although it is not the only one. There are more nano-delivery systems with advanced targeting capabilities being developed and utilized.

#### 3.1.3. Stimuli-responsive drug-controlled release

Conventional nano-formulations tend to release drugs continuously as they travel to their target sites, either through active uptake or passive diffusion. This uncontrolled release can result in unintended exposure of healthy tissues to the drug, potentially causing toxic side effects.

While ligand-modified nanostructures achieve spatial targeting of tumors, responsive nanostructures enable intelligent drug release to enhance cellular uptake in a controlled manner, both temporally and spatially. Stimuli-responsive nanostructure design mainly relies on internal and external stimuli. Internal stimuli mainly refer to the tumor microenvironment biomarkers, including acidic or hypoxic conditions, reactive oxygen species (ROS), specific enzymes, and glutathione (GSH). External stimuli, including near-infrared light (NIR) and magnetic fields, are commonly used to achieve controlled drug release^76^. Responsive boron-containing nano-systems used in BNCT include GSH-responsive nano-micelles^47^, thermo-responsive polymer micelles^49^, as well as chitosan nanoparticles^77^, acid-responsive^50^, and enzyme-responsive nanostructures^78^, as showed in Table 2.

For instance, Hao Quan *et al.* developed a micelle with a dual-responsive shell and core structure for precise boron delivery. The micelle was formed by HA shelled around the surface of positively charged o-carborane tagged by the cell-penetrating peptide TAT. The micelles can target tumor sites due to the EPR effect and HA's ability to bind to CD44. When HA enters the acidic tumor microenvironment, it is rapidly degraded by hyaluronidase (HAase), which is highly expressed in the tumor microenvironment. This degradation exposes the TAT, thereby enhancing the tumor cell membrane permeability of the micelles and facilitating responsive boron uptake^50^.

Besides, several types of magnetic nanoparticles have been designed for their potential application in BNCT. While magnetic targeting experiments have not yet been conducted, these nanoparticles have the potential to utilize magnetophoretic forces to accumulate boron drugs specifically at the tumor site, establishing a foundation for magnetically targeted delivery of boron nanoparticles.

### 3.2. Increase boron loading capacity and safety (Novel development trend)

Several tumor-targeting design concepts are illustrated above, which successfully increase the boron content at the tumor site. However, nano-systems still have major problems translating to clinical practice. On the one hand, inefficient boron delivery to the tumor will significantly impact the clinical treatment outcome. The emergence of boronizing nanocarriers doubles the boron load several times. On the other hand, biotoxicity and immunogenicity are real concerns that most nanoparticle platforms cannot be rendered completely non-toxic. Many biocompatible drug carriers, such as liposomes and micelles, have been designed to safely deliver boron. Biomimetic nanocarriers are another novel option for lowering biotoxicity and immunogenicity.

#### 3.2.1. Boronizing nanocarriers

The efficient delivery of boron is crucial for BNCT. However, whether using boron drugs or traditional boron-delivery nano-systems, the boron content in each dosing unit is limited. And after clearance by systemic circulation, the amount of boron that ultimately reaches the tumor site is significantly reduced. To meet the boron content requirements for BNCT, the dosage of boron drugs must be increased, raising the risk of toxicity associated with high drug concentrations in the body. In recent years, researchers have proposed novel approaches for directly integrating boron into nanocarrier materials, significantly increasing boron's delivery capacity and therapeutic effect.

**Figure 3:**
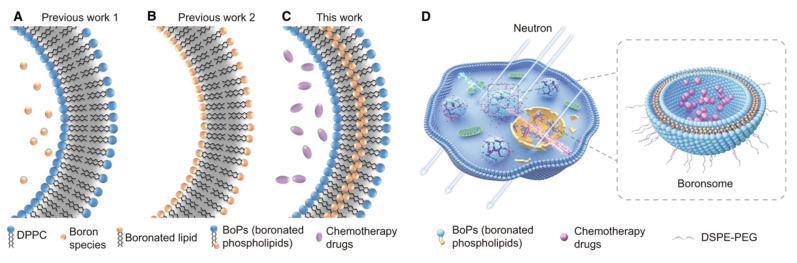
Schematic illustration of boronated liposomes for BNCT^103^, copyright 2022 Springer Nature. A-C Formulations of previous boronated liposomes (A, B) and boronated liposomes (C). D Boronsomes emit high-energy particles when irradiated by neutrons and release chemotherapy drugs sequentially to kill cancer cells.

For instance, researchers have developed a boron-containing liposome (boronsome) by directly incorporating boron into the phospholipid bilayer. This design not only increases the boron content but also enhances the stability and biocompatibility of the nanovesicles. The results show that boronsome exhibit high selective accumulation and prolonged retention in the tumor, thereby improving the efficacy of BNCT(Figure 3)^103^. In another study, lipoic acid-boronophenylalanine (LA-BPA) derivatives were used to develop multifunctional nanovesicles. By targeting SA on the surface of tumor cells through phenylboronic acid groups, these nanovesicles achieved the targeted delivery of boron to tumor cells. The boron concentration in the tumor reached 93.3 ppm, which is significantly higher than that in normal tissues. This design not only enhances the boron delivery capacity but also employs a GSH-responsive release mechanism to facilitate efficient drug release within tumor cells^98^.

Both of these nanocarrier systems demonstrate high boron content, excellent tumor targeting, and biocompatibility. These innovative approaches not only provide new strategies for the clinical application of BNCT but also offer important technological support for multimodal cancer treatment and precision medicine.

#### 3.2.2. Biomimetic nanocarriers loaded with boron

Traditional inorganic and organic nanocarriers face major challenges in clinical translation due to their immunogenicity and toxicity, which lead to extensive clearance by the reticuloendothelial system upon administration. In contrast, biomimetic nanocarriers have emerged as a promising alternative, offering enhanced biocompatibility and biological functions analogous to those of cells, which have garnered significant attention for drug delivery systems. Currently, extracellular vesicle (EV)-based nano-systems and cell membrane encapsulation are the most prevalent techniques in biomimetic cellular nanocarrier applications^104^. Furthermore, endogenous proteins have also been identified as a viable type of biomimetic nanocarrier^105^. The biomimetic nanocarriers loaded with boron have been listed in Table 3. However, as a rising star in the field of drug delivery, biomimetic nanocarriers remain underexplored in BNCT.

##### 3.2.2.1. Extracellular vehicles (EVs)

Extracellular vehicles (EVs), composed of lipid bilayers containing transmembrane proteins and soluble cytoplasmic components, are well-established mediators of intercellular communication. Exosomes are the most extensively studied EVs. They are cup-shaped vesicles with diameters raging from 30 to 100 nm. Their nanoscale size and ability to selectively target tissues make them ideal vehicles for delivering therapeutic agents. In addition, some exosomes have been shown to exhibit immunomodulatory effects^117^. Exosomes from HeLa cells have been studied as boron drug carriers in BNCT. The surface of EV was modified with hexadecyl oligoarginine (R16) peptide to enhance actin-dependent megacellular uptake. *In vitro* studies have showed that EV-encapsulated boron nano-systems exhibit higher anticancer activity than unencapsulated systems^109^. However, *in vivo* evaluation of these nanocarriers remains to be conducted.

##### 3.2.2.2. Cell membrane-encapsulated nanoparticles

Cell membrane-encapsulated nanoparticles are engineered by enveloping the synthesized nanoparticles with a natural cell membrane. The cell membrane, as a communication tool with the external environment, is crucial for cellular function. By mimicking the biological functions of the cell membrane, cell membrane-encapsulated nano-systems can achieve prolonged circulation *in vivo*, tumor homing, cancer cell targeting, vaccine delivery, and other related functions, thereby significantly enhancing nanoparticle stability^104^. Currently, cell membranes derived from erythrocytes, leukocytes, tumor cells, and bacterial bilayer membranes are primarily used to encapsulate nanoparticles. Researchers have prepared erythrocyte membrane-coated boron nitride nanoparticles (BN@RBCM), which significantly reduced the systemic toxicity of BN^111^. Additionally, a recent study has explored the encapsulation of boron nitride nanoparticles (BN) and doxorubicin (DOX) within a cancer cell membrane (CCM), forming a biomimetic nano-system denoted as CM@BN/DOX. The CCM-coated nanoparticles demonstrated good biocompatibility, strong targeting ability, and enhanced homogeneous tumor eradication^112^.

##### 3.2.2.3. Endogenous proteins derived nano-system

Endogenous proteins-derived nano-system is the third type of biomimetic structure, with albumin and ferritin being the most extensively utilized due to their low immunotoxicity. Classical albumin-paclitaxel nanoparticles have been clinically approved for tumor therapy, making these nanocarriers highly promising for various applications^105^. Currently, structural designs such as boron-albumin conjugated nanoparticles^113^,^115^,^116^,^118^ have been developed, which significantly enhance the stability and biocompatibility of boron drugs. Additionally, an archaea-derived protein has been engineered into a nanotube structure known as RHCC-NT, which is capable of stably encapsulating carbon borane within its lumen. In *in vivo* experiments, endotoxin-purified protein nanotubes elicit minimal immune response and are efficiently internalized by cells, accumulating in the perinuclear cytoplasm, resulting in a strong and effective neutron therapy-induced cytotoxic effect^106^.

## 4. Innovative Delivery Strategies Overcoming BNCT Bottlenecks

Boron-delivery systems continue to confront critical bottlenecks. Insufficient boron content in the tumor, as well as unfavorably low tumor-to-normal tissue and tumor-to-blood boron ratios, reduces the therapeutic effect. Tumor heterogeneity hinders the accumulation of nanoparticles, and dense solid tumors cannot be uniformly penetrated by relying solely on the EPR effect. The long-term biodistribution and metabolic pathways of nano-boron drugs are still inadequately studied, and potential biosafety risks require comprehensive preclinical evaluation. Although boron delivery systems have been supported by the latest research results in the field of pharmacy in terms of materials and structures, as outlined above, a growing number of researchers are now pursuing new delivery strategies that can appropriately correct the above deficiencies and achieve more clinically transformative breakthroughs.

### 4.1. Cross the blood-brain barrier (BBB) and blood-tumor barrier (BTB)

Given the high application status of radiotherapy in the treatment of cerebral tumors, BNCT also holds significant promise. However, the prerequisite for effective BNCT is the delivery of boron drugs across the BBB and blood-tumor barrier BTB, which remains a considerable challenge. Currently, two innovative studies have addressed this challenge by developing novel strategies to enhance the delivery of boron drugs to brain tumor sites.

In one study, Ching-Hsiang Fan *et al.* employed microbubbles as a delivery system. These microbubbles interact with the ultrasound energy generated by transcranial focused ultrasound (FUS) to enhance acoustic effects and microfluidic dynamics. This interaction generates mechanical stress that activates transient tight junction deformations, thereby noninvasively and reversibly opening the BBB/BTB to facilitate enhanced drug delivery to brain tissue. The study achieved a high tumor-to-normal brain ratio (T/N ratio) of 4.4, setting the stage for the realization of BNCT in brain tumors^119^.

In another study, the authors exploited the transient disruption of the BBB by intravenous mannitol. Mannitol administered intravenously 10 minutes before the administration of the designed boron-containing nanoparticles causes a temporary disruption of the BBB that lasts 10-60 minutes, ensuring that the nanoparticles penetrate the brain^94^.

### 4.2. Balance the drug clearance and retention

The development of effective boron drug delivery systems faces a dual challenge: boron drugs must be rapidly cleared from the bloodstream and normal tissues to minimize systemic toxicity, while simultaneously achieving sufficient accumulation in tumors during irradiation. These requirements are frequently contradictory, as the prolonged circulation times required for tumor accumulation can cause off-target accumulation in organs such as the liver and spleen, resulting in negative side effects. To address these challenges, new delivery strategies have been developed to enhance tumor retention while ensuring rapid clearance from non-target tissues.

#### 4.2.1. Transform of nano-scale

Irene V.J. Feiner *et al.* designed an ultra-small boron carbon dot nanoparticle. Trastuzumab, a monoclonal antibody with high tumor-targeting specificity, was functionalized with trans-cyclooctene (TCO) and administered. Subsequently, tetrazine (tz)-modified ultra-small boron carbon dot nanoparticles were introduced. These dot nanoparticles were enriched in tumor tissues through the orthogonal reaction of tz and TCO. Because of their small size, nanoparticles not captured by trastuzumab were quickly cleared from the body, resulting in rapid clearance of boron drugs while retaining them in tumors^120^.

**Figure 4:**
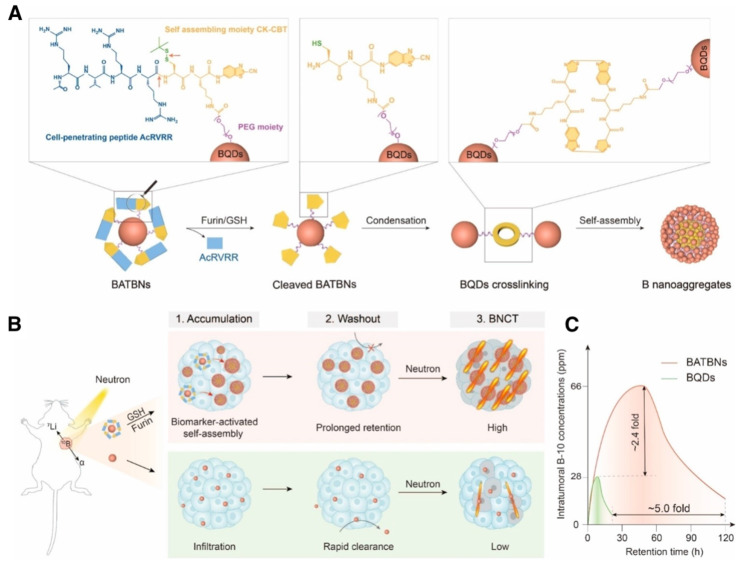
Schematic illustration of biomarker-activated size-transformed boron nanosensitizers for enhanced BNCT^121^, copyright 2024 Wiley-VCH GmbH. (A) Molecular mechanisms of the size transformation of small-sized boron nanomedicine into larger particle size nanoassembly. Red arrows indicate the sites of furin/GSH cleavage. (B) Schematic illustration of the intratumoral boron accumulation behavior of BATBN and size-fixed BQDs, and their BNCT efficiency. (C) The intratumoral boron concentration and retention time were significantly enhanced by BATBNs compared to BQDs.

Liping Li *et al.* made a smart boron nanosensitizer (BATBN) comprising an ultrasmall boron quantum dots (BQD) core conjugated with CPPs (Figure 4). The small size of the BQD core and CPPs facilitates the deep tumor penetration. Furin and GSH in TME induce the cleavage of CPPs, exposing disulfide bonds that trigger the self-assembly of BQDs. This process results in an increase in nanoparticle size, thereby optimizing boron retention within the tumor^121^.

#### 4.2.2. Boron drugs' antidote

In another innovative approach, phase-transformation lysozyme (PTL) was utilized to encapsulate boron carbide nanoparticles (BNNPs) for drug delivery. This method protects the BNNPs from hydrolysis during circulation, thereby prolonging their circulation time and enhancing boron accumulation in tumors. Furthermore, to mitigate off-target accumulation, vitamin C was administered intravenously to detoxify BNNPs that had accumulated in major organs, such as the liver and spleen. This strategy facilitated the rapid removal of BNNPs from these organs, thereby minimizing their toxic effects^122^.

#### 4.2.3. Block the reverse transport of LAT-1

BPA is a widely used boron delivery agent that targets LAT-1 for tumor targeting in BNCT. However, BPA's short retention time in tumors limits its effectiveness in BNCT. Reportedly, this limitation is related to the reverse transport mechanism of amino acid transport proteins, including LAT-1, which exchanges intracellular BPA for extracellular amino acids such as tyrosine. This exchange is unfavorable for BPA accumulation within tumor cells^57^. One study utilized polyvinyl alcohol (PVA) to form a borate complex with BPA. This complex not only facilitated the internalization of BPA into the cell via LAT-1-mediated transport but also resulted in the accumulation of BPA in lysosomes, slowing down the reverse transport mediated by LAT-1 and leading to the long-term retention of BPA in tumor cells^123^,^124^. Additionally, the authors exploited the partial cationic properties of the PEG-PLys backbone to promote renal clearance and enhance the tumor-to-blood (T/B) ratio, thereby further improving the therapeutic efficacy of BPA in BNCT.

Furthermore, studies have shown that both D-BPA and L-BPA can be absorbed by tumor cells via the LAT-1 transporter. However, L-BPA has a higher transport efficiency than D-BPA, making it easier to efflux through LAT-1's reverse transport. As a result, the authors created a PVA-D-BPA complex that significantly slows the reverse transport of LAT1. This strategy effectively increases the intracellular retention of BPA within tumor cells^125^.

### 4.3. Increase the enrichment of boron in the nucleus

The particle rays generated by BNCT through fission reactions exert cytotoxic effects within a range of approximately 10 μm, with their energy decaying exponentially as the distance increases^126^. Therefore, reducing the distance between ^10^B and the nucleus (ideally within 5 μm) would enhance the efficacy of α-particle-induced DNA double-strand breaks, thereby augmenting the tumoricidal capability of BNCT^59^. Therefore, the nucleus-positioning boron delivery system is highly anticipated. However, many nanocarriers remain extracellular or cytosolic and fail to achieve precise perinuclear or nuclear localization due to lysosomal clearance and their large diameter, which may reduce the local efficacy of high-LET particle damage and contribute to clinical recurrence and metastasis. An accurate and efficient nuclear-targeted delivery strategy still remains to be solved.

Several studies have demonstrated that various nano-systems can localize around or be internalized by the cell nucleus. These nano-systems include mesoporous silica nanoparticles^58^, boronated porphyrin-containing polymeric nanoparticles^48^, phenylboronic acid-modified polymeric nanoparticles^59^, RHCC protein helical nanotubes^106^, and multifunctional liposomes containing DOX-grafted carbaboranes^93^. Achieving precise delivery of boron drugs to the tumor cell nucleus could potentially reduce the required dose while maintaining therapeutic efficacy, thereby minimizing the toxic side effects associated with the systemic boron burden. For example, using DOX's ability to transverse the nucleus, carborane was introduced into the nucleus, resulting in boron aggregation^93^.

The primary strategies for achieving nuclear delivery of drugs include the use of nuclear localization signal (NLS) peptides and aptamers. The NLS is a specific protein domain that functions as a signal fragment to interact with nuclear import receptors, thereby mediating the transport of proteins from the cytoplasm to the nucleus. Aptamers, on the other hand, are short single-stranded DNA or RNA molecules that can bind to molecular targets with high affinity and specificity. Among these, the AS1411 aptamer serves as a ligand for nucleolin, facilitating nuclear transport^127^. However, since the NLS was modified on the boronated porphyrin in 2005^128^, no research has emerged regarding the use of NLS for the nuclear-targeted delivery of boron in the past two decades. And there are no reports about aptamers being used in boron's nuclear delivery. Besides, the majority of boron delivery systems that exhibit nuclear or perinuclear localization have merely observed this phenomenon without elucidating the underlying nuclear targeting mechanisms. Consequently, the reproducibility of these systems remains a matter of concern. Therefore, the development of boron delivery systems that specifically target the cell nucleus represents a valuable direction for future research.

### 4.4. Intra-arterial and intra-tumoral administration of boron drugs

Intra-tumoral administration is a direct method for delivering effective components to tumor sites, thereby achieving a high local drug concentration. Intra-arterial administration infuses the anti-tumor agents into the feeding artery of the tumor, which has been well-established in the treatment of liver cancer. These two administrations enhance drug bioavailability at the target site while simultaneously reducing systemic exposure to the therapeutic agents.

Intra-arterial administration of boron drugs has already been explored in BNCT of head and neck cancers^129^,^130^, brain tumors^131^, and liver cancer^132^,^133^. Early in 2006, researchers compared the intra-arterial (IA) and the intravenous (IV) delivery of boron drugs for recurrent head-and-neck tumors. The IA route achieved higher boron concentrations in the tumor, with one patient achieving a complete response (CR) and three showing a partial response (PR), compared to two PR, three stable cases, and one progressive disease (PD) in the IV group^129^. Hironobu Yanagie *et al.* used a 10B-shielded water-in-oil-in-water emulsion for intra-arterial injection in a liver cancer model, reporting boron concentrations of 61.7 ppm in the tumor, 4.3 ppm in normal liver tissue, and 0.1 ppm in blood^132^,^133^. These findings demonstrate that intra-arterial delivery can effectively concentrate boron drugs in tumors, enhancing efficacy and reducing toxicity.

Intra-tumoral administration has not yet been trialed in BNCT; however, it has been used for a broad range of agents in cancer immunotherapy, with encouraging clinical trial results^134^,^135^. Given its ability to achieve high local drug concentrations within the target tumor, intra-tumoral administration holds significant potential for enhancing the efficacy of BNCT.

Nevertheless, both intra-arterial and intra-tumoral drug administration remain underdeveloped in clinical practice, primarily due to their high technical thresholds and complex operational procedures. Intra-arterial administration requires the expertise of specialized surgeons, while intra-tumoral administration demands precise image guidance. Moreover, the anatomical location and depth of tumor growth influence the specific operational methods of drug delivery. Additionally, intra-tumoral administration is affected by tumor heterogeneity and the high interstitial fluid pressure within the tumor microenvironment. Therefore, the development of intra-arterial and intra-tumoral administration in BNCT in the future will require the collaborative efforts of radiation oncologists, surgeons, radiologists, and other healthcare professionals.

## 5. Synergistic Multifunctional Boron Delivery System

In addition to the vertical development of boron delivery systems to become more accurate, more abundant, and safer through the above means, researchers have increasingly focused on the horizontal development of multifunctional delivery systems that integrate BNCT with other therapeutic or diagnostic modalities. To meet the demand for a comprehensive treatment model in the clinic, a boron delivery system that combines chemotherapy, immunotherapy, gene therapy, photodynamic therapy (PDT), photothermal therapy (PTT), and other therapeutic approaches have begun to emerge. Furthermore, to achieve simpler, more reliable, and clinically applicable methods for the determination of boron content in tumors, the development of visualized theranostic boron delivery systems has also become a key focus.

### 5.1. Combination with chemotherapy

Various studies have used nanostructures to co-deliver boron and chemotherapeutic drugs, taking advantage of their favorable drug-carrying capabilities. Consequently, a combined BNCT and chemotherapy treatment approach has been successfully achieved.

Our team has successfully conjugated the chemotherapeutic agents DOX^93^ and paclitaxel (PTX)^92^ with carborane, respectively, to construct a multifunctional nano-system that integrates chemotherapy and BNCT. The RGD peptide was used to achieve tumor-targeting delivery, thereby enhancing the therapeutic efficacy of BNCT. Furthermore, BPA has been used as a boron source to form core-shell nanostructures for the delivery of methotrexate, a poorly water-soluble folate-based antimetabolite anticancer medication, in the treatment of glioblastoma multiforme^77^.

Some studies have demonstrated that neutron irradiation can also serve as a stimulus for drug-responsive release. Liping Li *et al.* designed a boron nanosheet DOX@BNNS with surface-loaded DOX for the treatment of triple-negative breast cancer. The study found that the nanosheet exhibited strong tumor-targeting capability. Additionally, the research revealed that neutron irradiation and the tumor's acidic environment can trigger the controlled release of DOX in the nano-system. This led to a significant improvement in the therapeutic efficacy of triple-negative breast cancer *in vitro* and *in vivo* and reduced the systemic toxicity associated with chemotherapeutic drugs^136^.

### 5.2. Combination with immunotherapy

Cancer immunotherapy has emerged as an advanced therapeutic modality, garnering significant attention for its ability to harness the immune system to combat cancer. Recently, the integration of immunotherapy with radiotherapy has become a focal point of research, with numerous global clinical trials underway in specific cancer types. However, the development of nano-delivery systems that combine immunotherapy with BNCT remains in its nascent stages, with only a limited number of studies having been conducted thus far.

**Figure 5:**
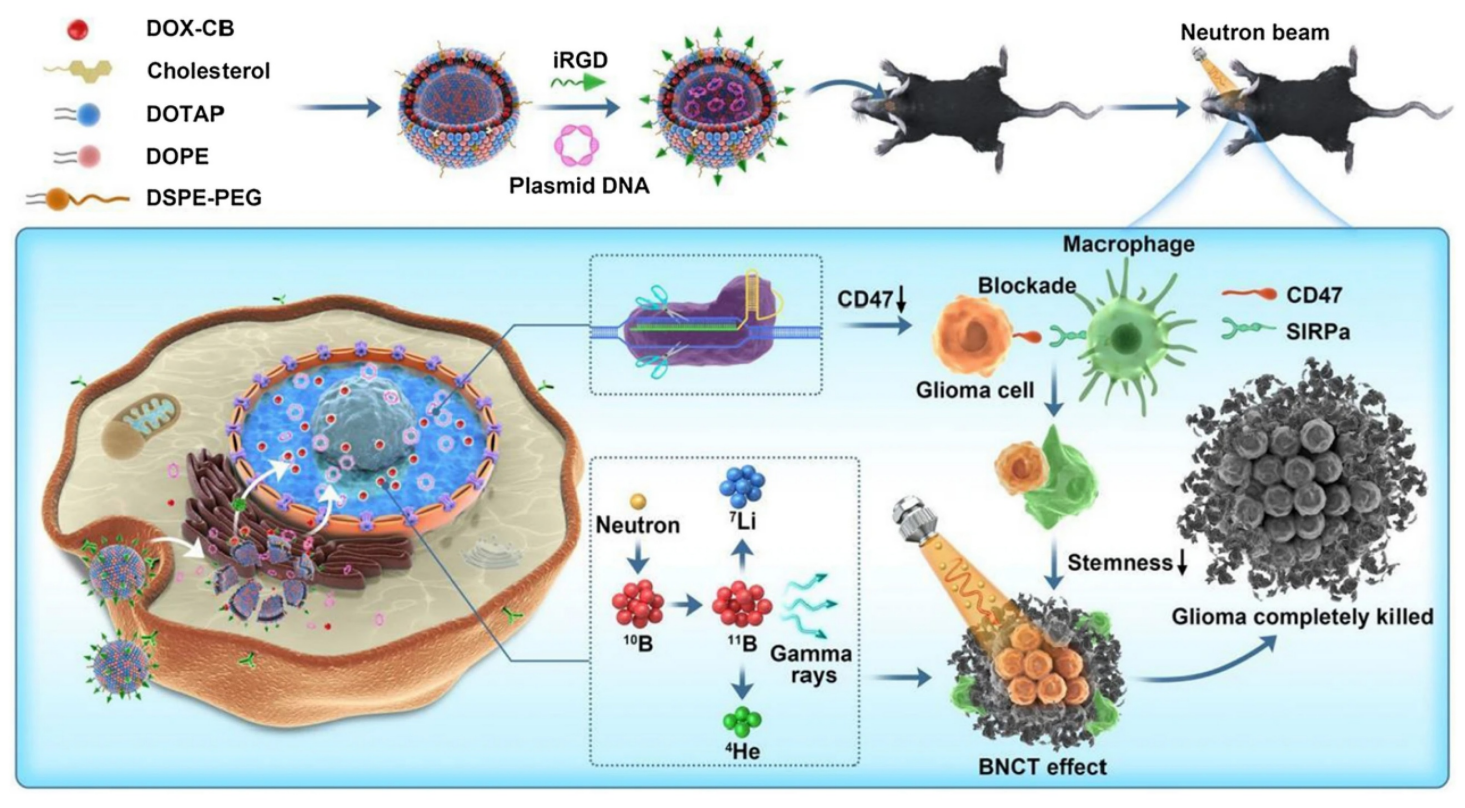
Schematic illustration of therapeutic mechanism of DOX-CB@lipo-pDNA-iRGD^93^, copyright 2022 Springer Nature.

Our team investigated a triple therapy nano-delivery system combining immunotherapy, chemotherapy, and BNCT, in which integrin-related protein CD47 was selected as the target for combination immunotherapy (Figure 5). CD47-blocking immunotherapy can effectively activate macrophage-mediated phagocytosis, promote adaptive immunity, and reduce the risk of recurrence. In our study, we employed cationic liposomes to deliver a CD47 gene-targeted CRISPR-Cas9 knockout plasmid for immunotherapy. In an *in vitro* study, CD47 expression in transfected GL261 cells was significantly reduced, leading to a marked enhancement of macrophage phagocytosis. In *in vivo* studies, this nano-system significantly improved anti-tumor efficacy, reduced the recurrence rate, and prolonged survival time^93^.

A new immunotherapeutic strategy utilizing inactivated Japanese enveloped hemagglutinating virus (HVJ-E) as a gene delivery vector has garnered considerable attention. Previous studies have demonstrated that the membrane fusion ability of HVJ-E activates anti-tumor immunity by inducing the expression of interleukin (IL)-6 and chemokine (CXCL)-10 in dendritic cells, thereby facilitating the elimination of cancer cells. The viral RNA genomic fragment of HVJ-E activates the retinoic acid-inducible gene I (RIG-I) signaling pathway and upregulates tumor necrosis factor-associated apoptosis-inducing ligand (TRAIL) and Noxa. However, as a vector, the virus is prone to membrane fusion with erythrocytes, leading to hemolysis. Therefore, a novel boron-containing biocompatible polymer modified on the HVJ-E surface was developed to significantly and synergistically enhance boron accumulation in tumor cells while providing immunotherapeutic effects^108^.

Shaohui Deng *et al.* developed a PD-L1 siRNA-loaded boron nanoparticle by forming phenylboronic ester bonds with the cis-diols of ribose groups at the ends of the siRNA. The nano-system can activate T-cell immunity while also suppressing DNA repair by inhibiting the expression of PD-L1 in tumor cells, resulting in more significant tumor immunogenic cell death. The results showed that the nano-system not only significantly inhibited the progression of primary and metastatic tumors but also demonstrated a strong abscopal effect^91^.

### 5.3. Combination with gene therapy

Boron drugs conjugated to nucleotides exhibit favorable bioavailability, and various mature synthetic methods for nucleotide-derived boron drugs have been developed^137^. Besides, the functional properties of nucleic acids can be used to achieve the dual effect of BNCT and gene therapy. For example, boron clusters incorporated in siRNA double strands enhance the resistance of siRNA to degradation without impairing its silencing activity^138^. Similarly, antisense oligonucleotides (ASOs) modified with boron clusters have been designed to regulate gene expression in disease. Researchers have designed a boron cluster-containing ASO that is able to inhibit the expression of EGFR and can be applied in BNCT concurrently^139^,^140^. Boron clusters have also been shown to increase RNase H activity in ASO silencing strategies, potentially enhancing ASO efficacy^141^.

However, the introduction of boron clusters could influence key properties of functional nucleic acids, such as lipid solubility, cell penetration, enzyme resistance, thermal stability, and binding affinity with complementary DNA and RNA. The effect of boron cluster introduction on the oligonucleotide depends on the specific site of incorporation, making this an important consideration in the design of nucleotide-complexed boron drugs.

### 5.4. Combination with PTT and PDT

The effectiveness of PTT is based on the photoabsorbent, which converts absorbed light energy into heat, thereby inducing thermal ablation of adjacent tumor cells. Research has demonstrated that PTT can enhance blood perfusion within tumor tissues, alleviating hypoxia and consequently augmenting radiosensitivity to a certain extent. And it can facilitate the further enrichment of boron drugs at the tumor site, which is advantageous for BNCT. Therefore, the integration of PTT and BNCT using nanomaterials represents a promising and synergistic therapeutic modality. In recent years, various nanomaterials, including gold nanostructures, carbon-based nanostructures, and copper-sulfur nanostructures, have been studied as photo-absorbers in PTT. Among these, gold nanoparticles garnered significant attention due to their low toxicity, high biocompatibility, and versatility in functional modifications, making them particularly suitable for use in PTT/BNCT co-delivery systems, and they have demonstrated promising results in this context^142^,^143^. Additionally, boron carbide nanoparticles, which inherently contain boron, have been reported as effective PTT nano-potentiators. These nanostructures enable the delivery of bimodal therapy within a single nanomaterial, thereby enhancing the therapeutic efficacy of combined PTT and BNCT^144^.

PDT is a cancer treatment using the biocompatible triple-state photosensitizers (PS) activated by light of a specific wavelength. The excited PS will produce either single-linear oxygen species that lead to direct cellular damage (Type II PDT) or ROS that lead to indirect cell death (Type I PDT). Common photosensitizers used in PDT include BODIPY, porphyrins, dihydroporphyrinols, phthalocyanines, and metal complexes. Specifically, dihydroporphyrinol^145^, porphyrin^146^, and Ru(II) or Ir(III) polypyridyl complexes[147]have been explored as photosensitizers in combination with boron drugs to create a combined therapeutic nano-delivery system for PDT and BNCT. For instance, a boronated porphyrin, Octa-anionic 5,10,15,20-tetra[3,5-(nido-carboranylmethyl)phenyl] porphyrin (H_2_OCP), which incorporates eight boron clusters on a porphyrin ring, has been designed. H_2_OCP accumulates intracellularly to a greater extent than BPA and BSH, making it a superior delivery system for BNCT. Moreover, it is a highly efficient photosensitizer with cytotoxic effects upon photoactivation^148^.

### 5.5. Theranostic boron drugs

The timing of neutron irradiation is determined by the ratios of boron concentration (T/B and T/N), which are typically estimated by measuring boron levels in the blood. However, this method is often inaccurate due to individual heterogeneity. Therefore, researchers have designed theranostic boron drugs that can accurately determine boron concentration, enabling the performance of neutron irradiation within the optimal time window. Currently, fluorescence, MRI, and PET imaging are the most commonly used methods.

Strategies for visualizing fluorescence range from using autofluorescent materials as boron drug carriers^87^,^101^,^145^,^149^ to grafting fluorescent chemical groups, such as Cy3, Cy5, and Cy7, onto boron carriers for imaging^150^,^151^. Fluorescent materials used as boron drug carriers include boron carbon oxynitride (BNCO), fluorescent gold nanoclusters, zinc gallate-based (ZnGa_2_O_4_:Cr^3+^), and dihydroporphyrin, etc. BNCO is an environmentally friendly phosphor which is non-toxic and non-rare-earth. Its photoluminescent properties make it suitable for imaging. Fluorescent gold nanoclusters (Au NCs) have emerged as an alternative to traditional fluorescent materials in biosensing, imaging, and optoelectronic display^152^. Persistent luminescent nanoparticles (PLNPs) based on zinc gallate groups (ZnGa_2_O_4_:Cr^3+^) have significant potential for bioimaging due to their ability to eliminate tissue autofluorescence and improve the signal-to-noise ratio. They can exhibit long-lasting phosphorescence in the near-infrared, which is suitable for *in vivo* bioimaging^153^. However, optical imaging is less applicable and is only used in animal studies. In the clinic, PET or MRI imaging is often used.

MRI is a widely used clinical imaging modality that has desirable intrinsic contrast in low-density tissue and better high spatial resolution. Unlike PET, which requires a radioactive tracer, MRI does not rely on ionizing or radioactive tracers, which can cause radioactive damage to tissues. MRI contrast agents, such as magnetic nanoparticles, can help distinguish between healthy tissues and lesions. In MRI, the two primary types of contrast agents are ferrous-containing magnetic materials and gadolinium (Gd) chelates. Among these, ferromagnetic nanoparticles have emerged as a research hotspot. Specifically, various ferromagnetic nanoparticles have been explored for the MRI localization of boron content, including manganese ferrite nanoparticles^143^, iron oxide nanoparticles^64^,^154^,^155^, and iron-boron composite nanoparticles^156^. Gd conjugate is a commonly used MRI contrast agent. Tissues with higher Gd uptake produce better image contrast in T1-weighted imaging. Meanwhile, ^157^Gd, similar to ^10^B, has a high thermal neutron capture cross-section and is a promising candidate for neutron capture therapy. A study was conducted on Gd atoms and boron cages co-encapsulated with gold nanoparticles in PLGA nanoparticles to visually examine the *in vivo* pharmacokinetics and tumor distribution. The nano-systems were found to be enriched at the tumor site, with a T/N ratio of 4.17^157^. In addition, MRI imaging has been performed with Gd-DTPA grafted into the mesopores of mesoporous silica nanoparticles^78^ or covered with silica coating^94^. Our team recently assembled Gd-DOTA and BPA-F into lipid nanocarriers, which enabled MRI visualization capability^158^.

PET is more sensitive than MRI^159^, but it has the disadvantage of requiring radiotracers for visualization, such as ^64^Cu and ^18^F. ^64^Cu is frequently bound to boron nitride, polymer, gold, or boronated porphyrin nanoparticles^48^,^122^,^142^,^146^,^160^ to be delivered to the tumor site along with boron, while ^18^F is chelated onto BPA for PET imaging^161^. The visualization of boron amounts in nano-systems has a useful role in providing delivery systems for BNCT and alternative diagnostic techniques that could facilitate future clinical trials.

Jae Hun Ahn et al. synthesized a boronic-acid-conjugated porphyrin and radiolabeled it with ^64^Cu, which has been proven to be a helpful boron-loaded theranostic agent for precise tumor imaging and BNCT^162^. Zhibo Liu et al. designed a bis-boron amino acid (BBPA) by replacing the carboxyl group of BPA with a trifluoroborate group (-BF_3_-). Radiological imaging is achieved through ^18^F-^19^F isotopic exchange at the B-F bond, allowing BBPA to accurately reflect the boron drug's *in vivo* distribution using PET imaging. Moreover, the additional boron atom introduced into the molecular structure of BBPA enables it to deliver twice the amount of boron compared to BPA^163^.

MRIs do not rely on the radioactive tracers, which are essential for PET-CT. But PET-CT is more sensitive than MRI. And in the application of BNCT visualization, the chemical bonding between ^18^F and BPA is tighter than the physical encapsulation of Gd or Fe with boron drugs, reflecting a closer relationship between contrast agent concentration and boron drug concentration. However, MRI is more accessible to patients and can be performed multiple times, allowing for more convenient real-time monitoring of boron drug accumulation in tumors.

The primary objective of the versatile boron drug delivery systems in BNCT is to facilitate combination therapy. While the majority of the studies reviewed above have demonstrated significant and effective outcomes, the true realization of combination therapy hinges on the widespread and mature clinical application of BNCT in the future. Despite showing promising tumor clearance in laboratory settings, the clinical scenario is considerably more complex. Therefore, there remains substantial scope for further research into the combination of BNCT with other therapeutic modalities.

Interdisciplinary cooperation is a crucial direction for the future development of BNCT. In the clinic, tumor treatment often requires a combination of multiple treatment methods tailored to the patient's specific condition. A variety of pre-clinical investigations have shown that interdisciplinary cooperation can enhance the anti-tumor effect. However, its clinical translation remains significantly hindered. On the one hand, BNCT itself is still restricted to early-phase clinical trials. On the other hand, the precise control of individual drug doses within a shared nano-platform remains technically challenging, and the safety remains to be defined. However, since it is a trend, the future will likely emerge with simpler, more standardized, and more definitive nano-delivery systems in BNCT.

## 6. Future Perspectives of BNCT

Most studies use combinations of off-the-shelf boron drugs and nanostructures in BNCT. However, the drug delivery bottleneck of BNCT has not been solved. To successfully promote BNCT for clinical use, collaboration among doctors, physicists, biologists, chemists, material scientists, and pharmacists is necessary.

From a physics perspective, a stable neutron source and the methods to control and determine the radiation dose are necessary. From a chemistry perspective, research is needed to develop a new structure for the boron drug and to modify the boron drug group to establish a chemical foundation. From a materials science perspective, materials that exhibit high biocompatibility, low toxicity, and high boron loading with tumor-affinity are desirable. From a pharmacist's perspective, clear pharmacokinetic studies of boron drugs, nanoparticles, and novel drug formulations or drug delivery systems are needed. From a biological perspective, exploring the biological pathways and mechanisms associated with BNCT is crucial for the subsequent development of this treatment, including its radiosensitivity, death pathways, changes in the immune environment, and abscopal effects.

**Figure 6:**
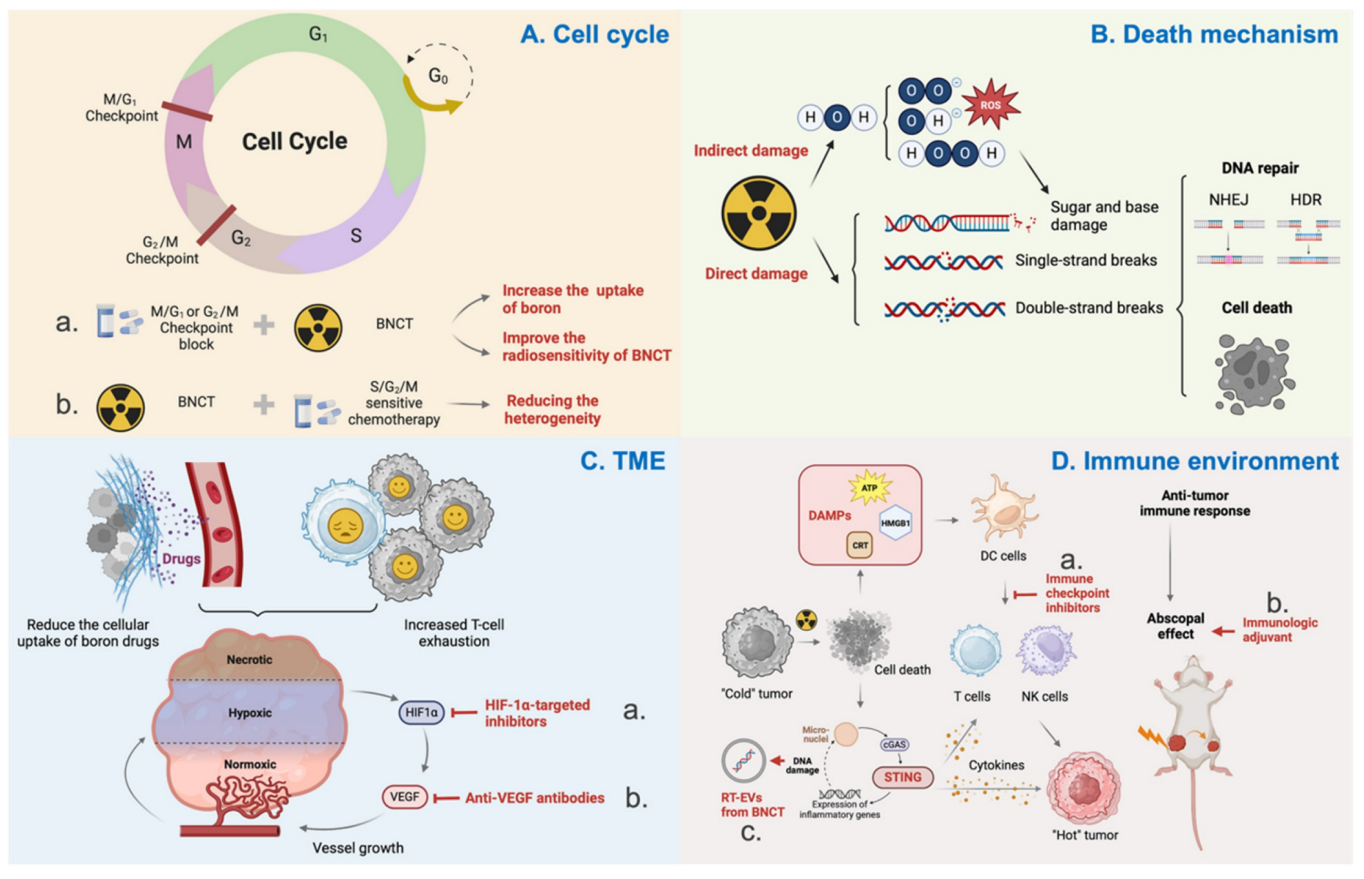
Summary of biological mechanisms underlying BNCT. A. The relationship of the cell cycle and radiosensitivity of BNCT. Checkpoint block will increase the uptake of boron and radiosensitivity (a). BNCT arrests the cell cycle at the G2/M to reduce the heterogeneity (b). B. Death mechanism of radiotherapy. Radiotherapy can induce indirect and direct damage to DNA. C. Tumor microenvironment influences the efficiency of radiotherapy. Anti-hypoxia (a) and anti-abnormal angiogenesis (b) can enhance the uptake of boron drugs and stimulate the immune response after radiotherapy. D. The immune response caused by BNCT. Immune checkpoint inhibitors will likely increase the immune response of BNCT (a); Immunologic adjuvants can increase the abscopal effect (b); The RT-EVs from BNCT with more damaged DNAs have greater anti-tumor immune response (c).

The structures, nano-systems, and delivery strategies of boron drugs have been discussed above. However, the biological mechanisms of BNCT have not been thoroughly explored, which is beneficial for the future development of boron drug delivery systems. This section will primarily elucidate the incomplete mechanisms of BNCT, providing insights for the design of boron delivery systems (Figure 6).

### 6.1. Cell cycle

The cell cycle can influence the radiosensitivity of cells, with the general order being G2/M > G1 > S^167^. This is related to the differences in DNA content and the DNA damage repair systems. Cells in the G2 phase have twice the DNA content of those in the G1 phase, which increases the initial yield of DNA double-strand breaks (DSBs) and, consequently, the probability of cell death. Additionally, the more accurate homologous recombination (HR) repair in the S phase reduces the radiosensitivity of cells during this phase.

The relationship between BNCT and the cell cycle can be summarized in two aspects:

1. BNCT generates high-LET radiation, which is more likely to arrest the cell cycle in the S or G2 phase compared to low-LET radiation. Studies have shown that BNCT can damage glioma stem/progenitor cells (GSPCs) that are resistant to X-rays and γ-rays, inducing cell cycle arrest at the G2/M phase and subsequently triggering apoptosis via the mitochondrial pathway^168^.

2. The cell cycle also affects the uptake of boron by cells. Cells in the S/G2/M phases can take up more BPA than those in the G0/G1/S phases, and this difference becomes more pronounced with increasing BPA concentration. Research suggests that this phenomenon may be related to L-type amino acid transporters^169^.

Considering the heterogeneity of tumor cells and the aforementioned theoretical basis, combining cell cycle-related chemotherapeutic agents to arrest cells in the S/G2/M phases can enhance the uptake of boron drugs and radiosensitivity in tumor cells^170^. Additionally, balancing the uptake of boron drugs across different cell cycle phases can also homogenize the cytotoxic effects of BNCT at the cellular level. Studies have already demonstrated that PVA-BPA can reduce the cell cycle dependency of boron uptake^171^.

### 6.2. Cell death mechanism

Radiotherapy kills cancer cells by damaging DNA, including ionization-induced sugar and base damage, single-strand breaks (SSBs), and DSBs^172^. In addition to the direct effects on DNA, charged particles can ionize water molecules surrounding DNA, generating highly reactive free radicals that cause indirect damage. For high-LET radiation, such as α particles and Li particles, direct damage predominates. In contrast, for low-LET radiation, the ratio of indirect to direct effects is approximately 3:1. Upon sensing DNA damage, cells initiate the DNA damage response (DDR) to maintain genomic integrity. The DDR is a complex network composed of multiple signaling pathways, ultimately inducing cell cycle arrest, DNA repair, and apoptosis^173^,^174^. The complex mixed radiation field is a characteristic of BNCT, which currently obscures the specific molecular pathways of tumor cell killing, the modes of tumor cell death, and the DNA repair patterns. Further exploration will help identify BNCT-specific radiosensitizers and, through the exploration of molecular pathways, uncover potential biological targets that may elevate the therapeutic efficacy of BNCT to a higher level.

### 6.3. Tumor microenvironment (TME)

Owing to the high oxygen consumption of tumor cells and the dysfunctional interstitial microvasculature, hypoxia is a common feature of the tumor microenvironment^175^. This hypoxic environment significantly limits the efficacy of conventional radiotherapy. BNCT-generated high-LET particle beams with low OER are less affected by hypoxia, thereby offering the advantage of overcoming the hypoxic conditions. However, it is regrettable that chronic hypoxia can reduce the cellular uptake of boron drugs and thus affect the efficacy of BNCT^176^,^177^. Moreover, hypoxia is often associated with increased T-cell exhaustion, which in turn suppresses the tumor immune response following BNCT. Studies have confirmed that hypoxia-inducible factor 1α (HIF-1α) can inhibit the expression of LAT-1 in hypoxic cells, thereby affecting the uptake of BPA^177^. BNCT's antitumor effects can be sensitized following treatment with HIF-1α-targeted inhibitors^178^. Therefore, overcoming hypoxia is also an excellent means of enhancing the efficacy of BNCT.

Hypoxia can induce the formation of abnormal blood vessels, and these malformed neo vessels, in turn, exacerbate hypoxia in TME. Additionally, the abnormal vascular morphology and reduced vascular density can decrease the perfusion and accumulation of boron drugs in tumors. Therefore, remodeling the tumor vasculature is a useful strategy to significantly improve the efficacy of BNCT. Currently, strategies for normalizing tumor vasculature mainly focus on blocking the expression of angiogenic factors. Bevacizumab, an anti-VEGF antibody, can normalize tumor blood perfusion and oxygenation during radiation therapy. It can also prevent radiation necrosis (RN) and symptomatic pseudoprogression (psPD) after reirradiation^179^. Additionally, some researchers have utilized vascular-targeting drugs to disrupt the abnormal vascular network, thereby facilitating the passive diffusion of boron drugs to quiescent cells and enhancing the effects of BNCT. Increased infiltration of immune cells into tumor tissues has also been observed following treatment^180^.

### 6.4. Tumor immune environment

Firstly, radiotherapy can promote immunogenic cell death, releasing damage-associated molecular patterns (DAMPs), which facilitate the priming and activation of immune cells. Secondly, activation of the STING inflammatory signaling pathway in cancer cells leads to the release of pro-inflammatory cytokines, resulting in the remodeling of the inflammatory microenvironment and the induction of the abscopal effect. Thirdly, the cytokines, stromal, immune, and vascular changes induced by radiotherapy reshape the tumor microenvironment, transforming "cold" tumors with limited immune cell infiltration into "hot" tumors infiltrated by lymphocytes. However, radiation therapy can also induce immune suppression by increasing inhibitory immune cells, including regulatory T cells (Tregs) and myeloid-derived suppressor cells (MDSCs), as well as the production of immunosuppressive cytokines^181^,^182^. Nevertheless, the relationship between radiotherapy and the immune system remains under continuous exploration, and the complex radiation sources of BNCT differ significantly from those of conventional radiotherapy, making its impact on the immune system largely unexplored^175^.

In the author's view, exploring the relationship between the immune environment and radiotherapy will promote the future development of BNCT in two key aspects: Firstly, immune checkpoint blockade (ICB) therapy is currently the most promising approach to immunotherapy. Preclinical and clinical evidence suggest that immune checkpoint inhibitors (ICIs) can be combined with radiotherapy to enhance therapeutic efficacy. The co-administration of ICIs and boron drugs targeting tumors may reduce immune-related adverse events in clinical practice, while potentially igniting the spark of an immune attack on tumor cells following BNCT. Secondly, since the abscopal effect was first described in 1953, it has been considered a promising frontier in cancer therapy, particularly for metastatic disease. The abscopal effect is primarily mediated through radiation-induced immunogenic cell death, which releases DAMPs and activates the immune system^183^. Studies have already observed the abscopal effect in xenograft mice when combining BNCT with immunotherapy^91^,^108^,^184^,^185^. The high-LET radiation generated by BNCT mainly induces direct DNA damage, with double-strand DNA damage activating innate immune signaling pathways. This suggests that BNCT has the potential to contribute to an abscopal effect. The integration of immunotherapy with advanced radiation techniques such as BNCT holds the potential to revolutionize treatment strategies.

Additionally, BNCT can also help with immunotherapy. The high-LET properties of alpha particles from BNCT can cause more DNA breaks and DNA oxidation, which resist nuclease degradation and accumulate in radiated tumor cell-derived extracellular vesicles (RT-EVs). It enhances antigen presentation, migration capacity, and cytokine secretion after being internalized by dendritic cells (DCs). These EV-educated DCs not only effectively inhibited primary tumor growth and metastasis formation but also elicited long-term immune memory^186^.

## 7. Conclusion

The authors contend that cost-effective boron delivery systems, characterized by their simple structure and high efficacy, are likely to hold a dominant position as BNCT progressively transitions into clinical practice. Small-molecule boron drugs may well carve out a bright future in this context. However, with the in-depth exploration of BNCT mechanisms, there is a significant potential for developing BNCT combination therapies that achieve synergistic effects far greater than the sum of their individual contributions. Therefore, boron nano-systems retain an indispensable value in this regard. Additionally, innovative delivery strategies that can effectively and simply address the bottlenecks encountered by delivery structures from an alternative perspective are essential and also need more brainpower.

## Figures and Tables

**Table 1:** Summary of boron nano-delivery systems based on ligand-receptor interactions

Target Point	Target Head	Drug Carrier	Boron Drug	Caner Species	T/B	T/N	Ref.
Folate receptor α	Folate	Boron nitride	Boron nitride	Glioma	/	/	79
		Boron carbide	Boron carbide	Cervical cancer	/	/	63
		Boron phosphate (BPO_4_)	Boric acid	Colon cancer; osteosarcoma	/	/	62
		Iron nanoparticles	Boric acid	Pancreatic cancer; cervical cancer; lung cancer	/	/	64
		Liposomes	Borane	Glioblastoma	/	/	53
		Polymers	Boric acid	Ovarian cancer	/	/	65
HER_2_	Anti-HER_2_ antibody	Boron nitride	Boron nitride	Ovarian cancer	390/24 h	170/24 h	80
		Liposomes	Carborane	Ovarian cancer	/	11/24 h	81
	61IgG	Silica nanoparticles	Boron cage-SH	Gastric cancer	/	41.05/12 h	73
EGFR	Anti-EGFR antibody	BPO_4_	Boric acid	Head and neck cancer	4.27/6 h	/	82
		GdB_6_	Boric acid	Head and neck cancer	4.18/24 h	/	83
		Liposomes	Boric acid	Glioma	/	5.6/24 h	84
	Cetuximab	Liposomes	BSH	Glioma	/	/	85
	Erlotinib	Liposomes	Carborane	Glioma	/	/	86
PSMA	PSMACR ligands	Boron carbon oxynitride (BCNO)	Boric acid	Astrocytoma	/	/	87
	ACUPA	Polymers	Carborane	Prostatic cancer	25/96 h	10/96 h	88
Integrin receptor αvβ3	cRGD	Gold nanoparticles (AuNPs)	BSH	Glioma	>50/6 h	>400/6 h	89
		Polymers	BSH	Melanoma	/	/	90
		Copolymers	Benzoborazole pendant	Breast cancer	5.2/24 h	18.1/24 h	91
		Chitosan nanoparticles	Carborane	Liver cancer	4.38/12 h	/	92
	iRGD	Liposomes	Carborane	Glioma	/	/	93
		Polymers	BSH	Lung cancer	14.11/24 h	19.49/24 h	75
	RGD-K	Silica nanoparticles	Boron	Astrocytoma	2.8/24 h	1.64/24 h in brain	94
LAT-1	BPA	Silica nanoparticles	BPA	Ovarian cancer	/	/	58
	Boronotryptophan derivatives	Polymers	Boronotryptophan derivatives	Glioma	/	170/50 min in U87; 8/1 h in LN229	95
Glucose-regulatory protein receptor	SP94 Peptide	Silica nanoparticles	Boric acid	Hepatic cancer	5.92/4 h	4.41/4 h	96
ASGPR	Trivalent galactosyl	Silica nanoparticles	Carborane	Hepatic cancer	/	/	96
CD44	Hyaluronic acid	Nanogels	BSH	Glioma	/	6/48 h in muscle; 2/48 h in brain	97
		Nanomicelles	Carborane	Breast cancer	3.58/12 h	/	50
Sialic acid	PBA	Polymers	BPA	Pancreatic cancer	4.1/6 h	6.7/6 h	98
		Polymers	PBA	Melanoma	/	/	59
		Copolymers	Fluorophenyl boronic acid	Cervical cancer; colon cancer	/	/	99
	BPA	Chitosan nanoparticles	BPA	Glioma	/	/	77

**Table 2:** Summary of boron nano-delivery systems based on stimuli-responsive release

Responsive Mechanism	Drug Carrier	Boron Drug	Caner Species	T/B	T/N	Ref.
H_2_O_2_ and GSH responsive	Boron nitride	Boron nitride	Breast cancer	5.5/12 h	41.5/12 h	100
MMP2/9 enzyme responsive	Silica nanoparticles	BSH	Chondrosarcomas	/	/	78
Acid responsive	Silica nanoparticles	Boric acid	Fibrosarcoma; melanoma	/	/	101
GSH responsive	Nanomicelles	BSH	Melanoma; breast cancer	/	/	47
	Copolymers	Benzoborazole pendant	Breast cancer	5.2/24 h	18.1/24 h	91
HAase and acid responsive	Nanomicelles	Carborane	Breast cancer	3.58/12 h	/	50
Light-responsive	Copolymers	BSH	Colon cancer	3/48 h	10/48 h	102
Thermo-responsive	Chitosan nanoparticles	BPA	Glioma	/	/	77

**Table 3:** Summary of biomimetic nanostructures

Drug Carrier	Boron Drug	Target Head	Target Point	Responsive Mechanism	Cancer Species	T/B	T/N	Multifuction	Other	Ref.
Protein/peptide nanotubes	Carborane	/	/	/	Breast cancer	/	/	/	Perinuclear localization	106
	BSH	/	/	/	Glioma	/	40/24 h	/	/	107
Virus	BSH	/	/	/	Liver cancer	/	/	Immunotherapy with virus	Abscopal effect	108
Extracellular vesicles	BSH	/	/	/	Glioblastoma	/	/	/	/	109
	Carborane	/	/	/	Colon cancer	63/24 h	420/24 h	/	/	110
Erythrocyte membrane	Boron nitride	/	/	/	/	/	/	/	/	111
Cancer cell membrane	Boron nitride	/	/	/	/	/	/	Chemotherapy with DOX	/	112
HSA	Boric acid	HSA	SPARC	/	Prostatic cancer; melanoma	/	5.94/3 h in prostatic cancer; 6.53/3 h in Melanoma	Fluorescence visualization with boron-containing carbon dots	/	113
	Dodecarborate	/	/	/	Glioma	/	/	Chemotherapy with gemcitabine	/	114
BSA	BSH	Hyaluronic acid	CD44	/	Breast cancer; lung cancer; liver cancer	/	/	/	/	115
	Dodecaborate	/	/	/	Oral cancer	1.6/19 h	4.7/19 h	/	/	116

**Table 4:** Summary of multifunctional boron nano-delivery systems

Multifunction	Function element	Drug Carrier	Boron Drug	Caner Species	T/B	T/N	Ref.
Chemotherapy combination	DOX	Boron nitride	Boron nitride	Breast cancer	2.4/24 h	/	149
		Liposomes	Carborane	Glioma	/	/	151
		Polymers	PBA	Pancreatic cancer	4.1/6 h	6.7/6 h	150
	PARP inhibitors	Liposomes	Carborane	Breast cancer	4/12 h	37/12 h	97
	Endostar	Polymers	BSH	Lung cancer	14.11/24 h	19.49/24 h	48
	Curcumin	Polymers	Boric acid	Ovarian cancer	/	/	83
	Methotrexate	Chitosan nanoparticles	BPA	Glioma	/	/	89
	PTX	Chitosan nanoparticles	Carborane	Liver cancer	4.38/12 h	/	166
Immunotherapy combination	anti-PD-L1	Nanomicelles	PBA	Melanoma	6.56/24 h	8.2/24 h	157
	PD-L1 siRNA	Copolymers	Benzoborazole pendant	Breast cancer	5.2/24 h	18.1/24 h	94
	CD47	Liposomes	Carborane	Glioma	/	/	78
PTT and PDT combination	PTT with boron carbide	Boron carbide	Boron carbide	Colon cancer	4.4/48 h	/	64
	PTT with AuNPs	AuNPs	Carborane derivatives	Gastric cancer	/	5.4/24 h	158
	PDT with chlorin	Iron nanoparticles	Bis (dicarbollide)	Glioblastoma	/	/	65
Fluorescence visualization	BCNO	BCNO	Boric acid	Astrocytoma	/	/	143
		BCNO	Boric acid	Breast cancer	3.5/24 h	/	156
	ZnGa_2_O_4_	Silica nanoparticles	Boric acid	Fibrosarcoma; melanoma	/	/	160
	Fluorescent gold nanoclusters	AuNPs	Carborane derivatives	Cervical cancer	/	/	142
	Cy3	Silica nanoparticles	Carborane	Hepatic cancer	/	/	103
	Cy7/Cy5	AuNPs	Dodecaborate	Glioma	/	/	146
	aza-BODIPYs	Nanogels	BSH	Glioma	/	6/48 h in muscle; 2/48 h in brain	95
	Boronated porphyrin; PET visualization with ^64^Cu	Polymers	Boronated porphyrin	Melanoma; breast cancer	2.5/24 h	6.54/24 h	73
CT visualization	CT/MRI visualization with GdB_6_	GdB_6_	Boric acid	Head and neck cancer	4.18/24 h	/	100
	AuNPs	AuNPs	BSH	Glioma	>50/6 h	>400/6 h	83
		AuNPs	Carborane	Osteosarcoma	/	/	149
MRI visualization	Gd	AuNPs	Thiol Boron cage-SH	Gastric cancer	/	4.17/24 h	151
		Silica nanoparticles	Boron	Astrocytoma	2.8/24 h	1.64/24 h in brain	150
		Silica nanoparticles	BSH	Chondrosarcomas	/	/	97
		Iron nanoparticles	Boric acid	Pancreatic cancer; cervical cancer; lung cancer	/	/	48
		Liposomes	BPA-F	Glioblastoma	4.29/6 h	7.82/6 h	83
		Polymers	Boric acid	Ovarian cancer	/	/	89
	MnFe_2_O_4_	Iron nanoparticles	Boric acid	Glioma	/	/	166
	Fe-B	Iron nanoparticles	Fe-B	Breast cancer; melanoma	/	/	157
PET visualization	^64^Cu	Boron nitride	Boron nitride	/	/	/	94
		AuNPs	Carborane derivatives	Gastric cancer	/	5.4/24 h	78
		Liposomes	Carborane	Breast cancer	4/12 h	37/12 h	64
		Copolymers	Carborane	Breast cancer	7.46/24 h	25.2/24 h	158
	^18^F	Polymers	Boronotryptophan derivatives	Glioma		170/50 min in U87; 8/1 h in LN229	65
SPECT visualization	I-123	AuNPs	Boron cage-SH	Gastric cancer	/	41.05/12 h	143
Chemodynamic therapy combination	Fe^3+^ (Fenton translation)	Boron nitride	Boron nitride	Breast cancer	5.5/12 h	41.5/12 h	156
GdNCT	Gd	GdB_6_	Boric acid	Head and neck cancer	4.18/24 h	/	160

## Abbreviations

BNCT): Boron neutron capture therapy
SBRT): Stereotactic body radiotherapy
LET): Linear energy transfer
RBE): Relative biological effectiveness
OER): Oxygen enhancement ratio
BPA): Boronophenylalanine
BSH): Sodium borocaptate
GBM): Glioblastoma multiforme
PDT): Photodynamic therapy
EPR): Enhanced permeability and retention
SA): Salivary acid
PBA): Phenylboronic acid
BPA-f): BPA-fructose
FR): Folate receptor
ASGP-Rs): Asialoglycoprotein receptors
HA): Hyaluronic acid
ADC): Antibody-drug conjugate
PDC): Peptide-drug conjugate
CPPs): Cell-penetrating peptides
CTPs): Cell-targeting peptides
ROS): Reactive oxygen species
GSH): Glutathione
NIR): Near-infrared light
HAase): Hyaluronidase
BCNO): Boron carbon oxynitride
AuNPs): Gold nanoparticles
boronsome): Boron-containing liposome
LA-BPA): Lipoic acid-boronophenylalanine
EV): Extracellular vesicle
BN): Boron nitride nanoparticle
DOX): Doxorubicin
CCM): Cancer cell membrane
BBB): Blood-brain barrier
BTB): Blood-tumor barrier
FUS): Focused ultrasound
TCO): Trans-cyclooctene
tz): Tetrazine
BQD): Boron quantum dots
PTL): Phase-transformation lysozyme
BNNPs): Boron carbide nanoparticles
PVA): Polyvinyl alcohol
NLS): Nuclear localization signal
IA): Intra-arterial
IV): Intravenous
CR): Complete response
PR): Partial response
PD): Progressive disease
PDT): Photodynamic therapy
PTT): Photothermal therapy
PTX): Paclitaxel
IL): Interleukin
ASOs): Antisense oligonucleotides
PS): Photosensitizers
DSBs): DNA double-strand breaks
HR): Homologous recombination
SSBs): Single-strand breaks
DDR): DNA damage response
TME): Tumor microenvironment
HIF-1α): Hypoxia-inducible factor 1α
RN): Radiation necrosis
psPD): Symptomatic pseudoprogression
DAMPs): Damage-associated molecular patterns
MDSCs): Myeloid-derived suppressor cells
ICB): Immune checkpoint blockade
ICIs): Immune checkpoint inhibitors
RT-EVs): Radiated tumor cell-derived extracellular vesicles
DCs): Dendritic cells
